# Vector competence of *Aedes albopictus* from Northern, Southeastern, and Southern Brazil for locally circulating East-Central-South African and Asian genotypes of Chikungunya virus

**DOI:** 10.1371/journal.pntd.0014522

**Published:** 2026-07-21

**Authors:** Maria Eduarda Barreto Resck, Daniel Cardoso Portella Câmara, Magda Clara Vieira da Costa-Ribeiro, Kelly de Oliveira Germano, Joaquim Pinto Nunes Neto, Maria Ignez L. Bersot, Dinair Couto-Lima, Flávia Barreto dos Santos, Barry Wilmer Alto, Nildimar Alves Honório

**Affiliations:** 1 Laboratório das Interações Vírus-Hospedeiros (LIVH), Instituto Oswaldo Cruz, Fiocruz, Rio de Janeiro, Brazil; 2 Scientific Computing Program, Oswaldo Cruz Foundation, Rio de Janeiro, Brazil; 3 Department of Basic Pathology, Post-graduation Program in Microbiology, Parasitology and Pathology, Federal University of Paraná, Curitiba, Paraná, Brazil; 4 Graduate Program in Parasitic Biology in the Amazon, Center for Biological and Health Sciences, State University of Pará, Belém, Pará, Brazil; 5 Department of Arbovirology and Hemorrhagic Fevers, Evandro Chagas Institute, Secretariat of Health and Environment Surveillance, Ministry of Health, Ananindeua, Pará, Brazil; 6 Laboratório de Mosquitos Transmissores de Hematozoários, Instituto Oswaldo Cruz, Fiocruz, Rio de Janeiro, Brazil; 7 Florida Medical Entomology Laboratory—FMEL, Institute of Food and Agricultural Sciences, University of Florida, Vero Beach, Florida, United States of America; 8 Fiocruz, Núcleo Operacional Sentinela de Mosquitos Vetores-Nosmove/Fiocruz, Rio de Janeiro, Brazil; International Centre for Genetic Engineering and Biotechnology, INDIA

## Abstract

*Aedes albopictus*, an invasive mosquito species prevalent across Brazil, serves as a crucial vector for numerous arboviruses, including Chikungunya virus (CHIKV). Following the introduction of Asian and East-Central-South African (ECSA) genotypes in 2014, Brazil has faced escalating CHIKV epidemics, underscoring the critical need to understand region-specific vector competence. Building upon previous research that indicated variable vector competence, this study evaluated the susceptibility and dissemination rates of *Aedes albopictus* populations from diverse Brazilian regions (Belém, Paraná and Rio de Janeiro) to locally circulating ECSA and Asian CHIKV genotypes. Body, leg (dissemination), and saliva (transmission) positivity rates were measured at 3, 5, and 13 days post-infection. Despite descriptive heterogeneity, there was no significant effect of time, population, or genotype on initial CHIKV infection rates in mosquito bodies, with over 80% of individuals infected as early as 3 days post-infection, suggesting highly permissive midguts. In contrast, CHIKV dissemination to the legs was significantly influenced by both time post-infection (showing progressive increase) and mosquito population (Paraná exhibited significantly higher rates than Belém), but not by virus genotype, indicating population-specific variations in systemic viral spread. Crucially, saliva positivity rates were consistently low across all tested populations and genotypes, being substantially lower than those observed in bodies and legs but showed no significant effect of time, population origin, or virus genotype on overall saliva positivity rates. Understanding these population-specific vector dynamics is critical for refining predictive models, accurately assessing regional outbreak risks, and developing public health interventions against CHIKV in Brazil.

## Author summary

The invasive mosquito *Aedes albopictus* is now established across all Brazilian states and serves as a potential vector for various pathogens, including Chikungunya virus (CHIKV). Since 2014, Brazil has faced simultaneous outbreaks of two viral lineages: the Asian and East-Central-South African (ECSA) genotypes. This study evaluated the “vector competence”—the biological ability to acquire and transmit a virus—of *Ae. albopictus* populations from three distinct regions: Belém (North), Rio de Janeiro (Southeast), and Paraná (South). Our findings reveal that the mosquito’s midgut is highly permissive to infection, with over 80% of mosquitoes becoming infected as early as three days, regardless of the viral genotype or the mosquito’s origin. While the virus disseminated to the legs more efficiently in mosquitoes from Paraná compared to those from Belém, the overall transmission potential remained low. Specifically, the presence of virus in the saliva was significantly lower than in the rest of the body, identifying the salivary glands as a major barrier to transmission. Understanding these region-specific interactions is vital for refining public health risk assessments and developing targeted strategies to control the spread of Chikungunya in Brazil.

## Introduction

In Brazil, *Ae. albopictus* was first detected in 1986 in Rio de Janeiro state, expanding its distribution to neighboring states, such as Minas Gerais and São Paulo [1,2]. The following year, it established itself in all states of the Southeast region and slowly dispersed throughout the country [1,3]. The first record of *Ae. albopictus* in southern Brazil occurred in 1996 in Paraná [4], and by 1999, *Ae. albopictus* had already been reported in 14 Brazilian states [5].

In April 2002, *Ae. albopictus* larvae were detected in ovitraps in the municipality of Medicilândia, in northern Brazil, marking the first record of *Ae. albopictus* in the state of Pará [6]. In 2002, the mosquito was already present in 20 of the 27 Brazilian states, except for Acre, Amapá, Ceará, Piauí, Roraima, Sergipe, and Tocantins [7]. Twenty-eight years after its first detection, the distribution of *Ae. albopictus* had extended to 24 of the 27 states in Brazil, with only Acre, Amapá, and Sergipe remaining [8]. By the end of 2019, the presence of this vector mosquito was confirmed in the municipalities of Sergipe and Amapá, and finally, in early 2023, in the state of Acre. Therefore, currently, *Ae. albopictus* is found in all 27 Brazilian states [9].

Although typically associated with areas of abundant vegetation and dispersed human populations, *Ae. albopictus* has also been found in transitional habitats where vegetation cover is relatively low [10–12], and in densely urbanized areas [13]. This invasive mosquito species is frequently captured outside houses, preferring the peridomestic to the intradomestic environment [11,14], and has adapted well to suburban and urban environments. In China and Italy, for example, it has already been described as the sole vector of arboviruses in urban areas [15,16].

The finding of *Ae. albopictus* inside Brazilian households where febrile cases of arboviruses have been reported clearly indicates that this species has a tendency towards domesticity, and despite not being considered as efficient a vector as *Ae. aegypti*, it may have epidemiological importance [13]. Field studies have established that, *Ae. albopictus* exhibits a broad host range in its feeding habits, being able to feed on the blood of several other vertebrates besides humans [17,18]. The successful introduction of *Ae. albopictus* on several continents, such as North America, Pacific Islands [19], South America [20], Africa, and Europe [21], is due to its generalized habitat and feeding needs, egg resistance to desiccation, adaptability to different climatic conditions, ability to live in human-dominated habitats [19,22], as well as competitive superiority in relation to resident species [23–25].

*Aedes albopictus* is a well-known and competent vector, capable of transmitting over 25 arboviruses. It’s been responsible for serious outbreaks of diseases like Chikungunya, Mayaro, Japanese encephalitis, Rift Valley, West Nile, and Sindbis viruses [26–31]. Beyond its established roles in horizontal transmission and its adaptability, an increasingly critical aspect of *Ae. albopictus*’s epidemiological significance lies in its capacity for natural vertical transmission of arboviruses. This mechanism, where the virus is passed directly from an infected female mosquito to her offspring, allows for the persistence of pathogens in mosquito populations even in the absence of infected vertebrate hosts, potentially contributing to the maintenance and re-emergence of arboviral cycles [32,33]. This phenomenon suggests a ‘silent circulation’ of arboviruses in populations of *Ae. albopictus*, indicating that green areas can serve as reservoirs, maintaining arboviruses not actively circulating in humans and thereby increasing the risk of future outbreaks (i.e., mediated by *Ae. albopictus* or other species such as *Ae. aegypti*). Furthermore, recent findings by Mbaoma et al. (2025) [34] reinforce the widespread occurrence of vertical transmission across various mosquito species, including *Ae. albopictus*, for a range of arboviruses like DENV, CHIKV, and ZIKV. Their work highlights that despite often being overlooked, vertical transmission significantly contributes to the dynamics of mosquito-borne arbovirus transmission and can influence outbreak patterns and endemism, emphasizing its crucial role in sustaining arbovirus transmission.

The invasive mosquito *Ae. albopictus* remains a significant public health concern in the Neotropics as well as other geographic realms, not only as a primary vector for epidemic arboviruses like dengue and chikungunya but also due to its potential role in the emergence of zoonotic diseases [8,31,35]. Previous studies have shown that *Ae. albopictus* can colonize and disperse into forest environments from urban edges, and its opportunistic feeding behavior allows it to feed on a wide range of hosts, including humans, domestic animals, and even wildlife [36–40]. This ecological flexibility in the behavior of *Ae. albopictus* as a potential ‘bridge vector’ at the urban-forest interface, facilitating the spill-over of enzootic pathogens from sylvatic cycles to human populations, thereby increasing the risk of novel arbovirus outbreaks in urban and peri-urban areas [39,41]. Understanding the vector competence of different geographic populations of *Ae. albopictus* for circulating arboviruses is therefore critical for assessing and mitigating the risk of disease transmission [28,42].

Chikungunya virus (CHIKV) is a mosquito-borne arbovirus that has seen a quick progression from tropical areas to subtropical regions and the Western Hemisphere [43–45]. This arbovirus is spread by invasive *Stegomyia* mosquitoes within the genus *Aedes*, and urban outbreaks have occurred worldwide, notably in areas where these mosquitoes are common. Chikungunya virus (CHIKV) likely originated in sub-Saharan Africa, where it was maintained in a sylvatic cycle involving wild primates and arboreal mosquitoes. Eventually, the virus shifted into an urban environment, where its transmission became sustained by mosquitoes that live near and interact frequently with humans [46]. Genetic evidence suggests that this urban transmission cycle began in East Africa and later spread to other global regions [47,48]. CHIKV is now categorized into three primary genetic lineages: West African, East-Central-South African (ECSA), and Asian. A notable sub-lineage of the ECSA genotype, termed the Indian Ocean lineage (IOL), has been linked to widespread outbreaks since it emerged in 2005 [48–50].

The simultaneous introduction of the Asian and ECSA genotypes of CHIKV in Brazil during 2014 initiated local transmission of the virus, with initial foci in the municipality of Oiapoque, Amapá (North), and Feira de Santana, Bahia (Northeast) [51,52]. Subsequent to this introduction, the ECSA genotype of CHIKV expanded among the Brazilian states, evidenced by recorded outbreaks in the Northeast [Bahia [53,54]; Alagoas [55,56], Piauí [57], Sergipe [58,59], Maranhão [60]], North [Roraima [61]], Southeast [Rio de Janeiro [62–65] and Minas Gerais, [66]], and Midwest regions [67]. Since 2016, Brazil has become the principal area of CHIKV epidemic activity in the Americas, experiencing the regular occurrence of outbreaks yearly [68]. In recent years chikungunya has continued to pose a significant public health challenge in Brazil, with a fluctuating yet concerning trend in reported cases and associated fatalities from 2023 to 2025. In 2023, the country registered 158,060 probable chikungunya cases, with 33 deaths under investigation and 122 confirmed deaths directly attributed to the virus. The situation worsened in 2024, seeing a notable increase to 265,545 probable chikungunya cases, alongside 68 deaths under investigation and a stark rise to 243 confirmed chikungunya-related deaths. In 2025, Brazil had recorded 127,919 probable chikungunya cases, with 51 deaths still under investigation and 123 confirmed fatalities. As of the 12^th^ epidemiological week of the current year (2026) Brazil has recorded 22,165 probable chikungunya cases, with 13 deaths still under investigation and 15 confirmed fatalities. This persistent burden underscores the ongoing need for robust surveillance, prevention, and control measures to mitigate the impact of this mosquito-borne disease on human populations [69].

The ECSA genotype’s expansion across Brazil, seemingly leading to the replacement of the Asian genotype in Roraima within the Amazon region, suggests a greater capacity for infectivity to the vector, facilitating its transmission and expansion of this genotype in Brazil [61,62,70]. In contrast, the CHIKV-Asian genotype’s presence in Brazil has predominantly been limited geographically to the state of Amapá in the North region [71].

The capacity of an insect vector to become infected and transmit a pathogen, termed vector competence, is often a highly specific characteristic determined by both genetic and non-genetic factors (e.g., environmental variability), and their interactions. However, it is crucial to recognize that vector competence is not a fixed trait within a species and can exhibit significant variation across different populations of the same vector species [72]. This quantitative characteristic is governed by the intricate interplay of genetic factors in both the transmitting insect and the virus it carries and is further modulated by environmental conditions [73–75].

Evidence from the analysis of complete viral genomes has revealed specific adaptive mutations in at least three independent instances, strongly suggesting that these genetic alterations have conferred a selective advantage for CHIKV transmission by *Ae. albopictus* [49,76–79]. This highlights the dynamic nature of virus-vector interactions and the potential for rapid evolutionary adaptation. In fact, studies on CHIKV have revealed that vector competence is influenced by the CHIKV genotype, the temperature during the virus’s development within the mosquito, and geographic variations within *Ae. aegypti* and *Ae. albopictus* mosquito populations, as well as distinctions between these two mosquito species [80–84]. Several studies have investigated the vector competence of *Ae. aegypti* and *Ae. albopictus* for different genotypes of chikungunya virus (CHIKV) across different regions of the world [28,42,49,79,80,85–92].

Both *Ae. aegypti* and *Ae. albopictus* are susceptible to infection and capable of viral dissemination when exposed to the East/Central/South African (ECSA) and Asian genotypes of CHIKV. However, the efficiency of this process varies according to specific viral strain and experimental conditions [85,87]. Given the demonstrated variability in vector competence for CHIKV, particularly in *Ae. albopictus*, understanding the factors that influence this trait is critical for evaluating chikungunya transmission and spread. While previous studies like Honório et al. (2018) [42] and Vega- Rúa et al (2014) [28] have initiated assessments of Brazilian *Aedes* populations for specific CHIKV genotypes (e.g., Asian genotype), a comprehensive understanding of how geographically distinct *Ae. albopictus* respond to locally circulating ECSA and Asian CHIKV genotypes remains crucial. Notably, the vector competence of *Ae. albopictus* populations can differ based on their geographical origin and the specific viral genotype involved in the infection. While the ECSA genotype of Brazilian CHIKV lacks the well-characterized E1-A226V mutation associated with enhanced adaptation in other regions, this does not preclude the existence of other relevant genetic changes that warrant further investigation [93]. Therefore, we evaluated the susceptibility and dissemination rates of *Ae. albopictus* populations from different Brazilian regions to locally circulating ECSA and Asian CHIKV genotypes. This comprehensive approach aims to provide critical insights into regional variations in vector competence, which are essential for robust risk assessment and targeted control strategies across Brazil.

## Materials and methods

### Ethics statement

All viral strains, *Aedes albopictus* populations and shipment used in this study were recorded in the Brazilian System for the Management of Genetic Heritage (SisGen A5D493C and RAAA843). The study protocol was approved by the Research Ethics Committee of the Oswaldo Cruz Institute (CEP FIOCRUZ/IOC) under approval number 75375223.6.0000.5248. Formal consent was not required as the study involved only viral strains and mosquito populations, with no direct participation of human subjects or collection of identifiable personal data.

### Mosquito collections and rearing

Brazilian *Ae. albopictus* populations used in this experiment were obtained through eggs collected in oviposition traps in 2021 and 2022. *Aedes albopictus* population from the Southeast region (Rio de Janeiro, RJ, 22°52’33.0"S 43°14’51.4"W) originated from the Manguinhos campus, Oswaldo Cruz Institute, Fiocruz. The North region (Pará, PA1°22’28.69"S 48°23’04.04"W) and South region (Paraná, PR, 25°25’49.0"S 49°18’13.8"W) *Ae. albopictus* populations were collected during routine entomological surveys and provided by the Evandro Chagas Institute, Pará, and Federal University of Paraná, respectively. These sites were selected because they span distinct Brazilian regions and biomes, capturing a wide range of environmental conditions and historical patterns of CHIKV endemicity.

Field-collected eggs were reared in pans containing 1 L of tap water (150 larvae per pan) to adulthood on a diet with approximately 1g of yeast, larvae were fed with the same quantity of yeast, every two days, until pupal development. Upon pupation, pupae were collected daily and placed in cages until eclosion after which adult mosquitoes were identified to species and separated to establish a low-passage lab colony of *Ae. albopictus*. The mosquitoes were kept in cages (31 cm wide x 32 cm long x 33 cm high) within an insectary maintained at 28 ºC with a 12-hour light/12-hour dark cycle. They were provided with a 10% sucrose solution via cotton wicks and received weekly blood meals from chickens (IACUC protocol 201003892) at UF-FMEL. *Aedes albopictus* populations from Rio de Janeiro, Belém, and Paraná used in the experiment were from 3rd (F3), 4th (F4), and 4th (F4) generations, respectively.

### Virus and mosquito oral infection

For experimental infection epidemic strains of CHIKV representing the genotypes currently circulating in Brazil were used. The PER160/H803609 strain (GenBank accession number KP164571.1), belonging to the Asian genotype, was isolated from the plasma of an infected patient from an epidemic that occurred in Pernambuco in 2014, and the BHI3741/H804705 strain (GenBank accession number KP164569.1), belonging to the ECSA genotype, was isolated from the serum of a patient during the epidemic that occurred in Bahia in 2014[52]. Both were provided by the Arbovirus and Hemorrhagic Fevers Section (SAARB) of the Evandro Chagas Institute, Pará.

For virus suspension preparation, African green monkey (Vero) cell monolayers were inoculated with diluted CHIKV stock at a multiplicity of infection (MOI) of 0.1 and incubated for one hour at 37 ºC in a 5% carbon dioxide atmosphere. These virological procedures were conducted at the UF-FMEL biosafety level 3 (BSL-3) facility, in accordance with established BSL-3 standard operating procedures. After the inoculation procedure, 20 mL of medium (composed of M199, 10% fetal bovine serum, 2% penicillin/streptomycin, and 2% nystatin (Mycostatin) was added to each flask, which were then incubated at 37 ºC under a 5% carbon dioxide atmosphere for 3 days.

Using an artificial membrane feeding system (Hemotek, Lancashire, UK) at 28 ºC for 1 hour, seven-to-ten-day-old adult females from each population were offered defibrinated bovine blood (HemoStat Laboratories, Dixon, CA, USA) containing freshly propagated CHIKV warmed to 37 ºC. The observed blood feeding rate was approximately 30%. The use of an artificial feeding system has the advantage of standardizing the viral dose across all mosquito populations compared to a live host model. Quantitative comparisons of vector competence measurements for two different chikungunya virus genotypes requires that mosquitoes ingest blood with similar viral titers which is much easier to control using the artificial feeding system than mosquitoes feeding on live hosts. Before and after each blood feeding trial, 1 mL aliquots of CHIKV-infected blood were collected and stored in 2 mL cryogenic vials (Millipore Sigma, Burlington, MA, USA) at −80 ºC. These aliquots were used to determine the viral titer of the infected blood, which was found to be 8.0 log_10_ ± 0.32 plaque-forming unit equivalents per mL (PFUe/mL) in the blood meals. We deliberately used a high-titer blood meal to ensure that enough mosquitoes developed saliva infections for assessing treatment effects. Using lower viral titers would have limited our ability to detect treatment-dependent differences because too few mosquitoes would exhibit saliva infection.

### Chikungunya virus dissemination and transmission

Following feeding trials, females were anesthetized with carbon dioxide and kept cool within a metal tray set on ice. Mosquitoes were sorted and thirty to forty fully engorged females were transferred to cages (0.47 L food containers with mesh lids) and held for 3-, 5- and 13-days incubation period until sample collections were tested to determine susceptibility to infection, disseminated infection, and transmission rates. To determine susceptibility to viral infection and disseminated infection, each mosquito body and legs, respectively, were homogenized (TissueLyser II sample disruptor; Qiagen, Germantown, MD, USA) in medium supplemented with fetal bovine serum and centrifuged before viral RNA isolation and detection by quantitative RT-PCR.

For transmission assays, females were deprived of sucrose, but not water, for 24 h and transferred to 37 mL plastic tubes (height x diameter: 8 by 3 cm) with removable mesh lids, containing cationic paper (Q) “Q-paper” (1 cm in diameter) soaked with honey dyed with blue food coloring (McCormick, Hunt Valley, MD, USA). Thus, individually held female mosquitoes were offered a sugary meal to induce female salivation to collect saliva samples. The visual detection of the food coloring through the crop allowed for determination of fed versus unfed individuals, as previously described in Honório et al. 2018 [42].

### Viral nucleic acid extraction and quantitative RT-PCR

RNA was extracted from individual samples by processing 140 μL of homogenate with the QIAamp Viral RNA Mini Kit (Qiagen, Valencia, CA, USA). The RNA was eluted in 60 μL of buffer, following manufacturer’s protocol. CHIKV viral RNA was quantified by RT-qPCR, using the SuperScript III Platinum RT-qPCR Kit (Invitrogen, Carlsbad, CA, USA) in a CFX96 thermocycler (Bio-Rad Laboratories, Hercules, CA, USA). Each reaction comprised of a master mix containing 10 μL of 2X Reaction Mix, 2.2 μL of diethyl pyrocarbonate (DEPC) treated water, 1.0 μL of forward primer (10 μM), 1.0 μL of reverse primer (10 μM), 0.4 μL SuperScript™ III RT/Platinum™Taq Mix, 0.4 μL of probe, and 5.0 μL of viral RNA template (25% of reaction volume) for a total volume of 20 μL. Negative controls consisted of a sham viral RNA template of DEPC-treated water or dilute stock virus, each using a volume of 5.0 μL. Each mosquito sample and controls were tested in duplicate. Each sample with a quantification cycle (Cq) value of <35 was determined as positive. The thermocycling conditions were as follows: 50 ºC for 30 min, 94 ºC for 2 min, 39 cycles at 94 ºC for 10 s and 60 ºC for 1 min, and 50 ºC for 30 s. The following sequences represent primers designed to target a nonstructural polyprotein gene (accession ID of transcript, KU365292.1): forward, 5′-GTACGGAAGGTAAACTGGTATGG-3′; reverse, 5′-TCCACCTCCCACTCCTTAAT-3′. The probe sequence was 5′-/56-FAM/TGCAGAACCC ACCGAAAGGAAACT/3BHQ_1/- 3′ (Integrated DNA Technologies, Coralville, IA, USA). These primers and probe have been used successfully in previous studies assessing vector competence of mosquitoes for CHIKV [42]. Viral titer quantification was determined using a standard curve with serial dilutions of CHIKV stock, in parallel with titration by plaque assays of the same virus dilutions, expressed as plaque-forming unit equivalents (pfue)/mL.

**Fig 1:**
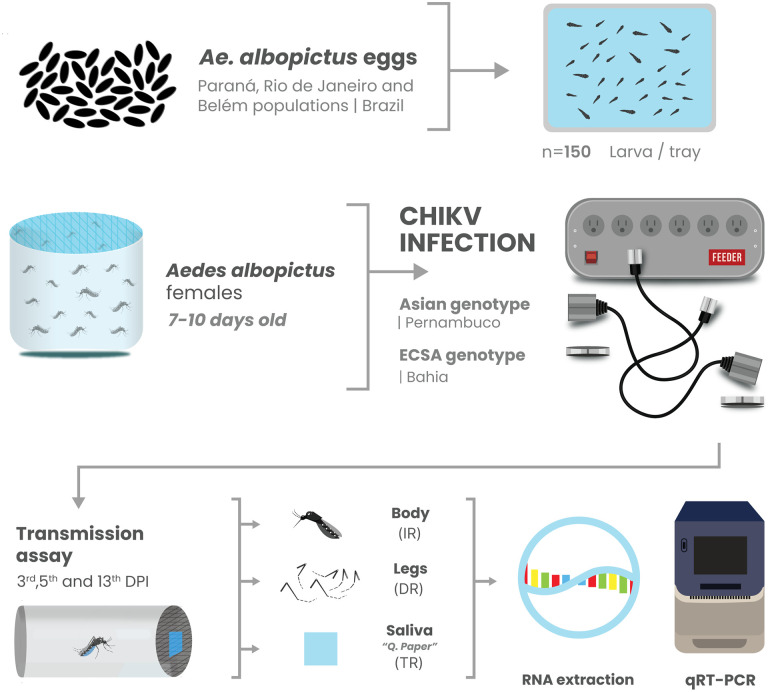
Schematic overview of experimental design. *Aedes albopictus* adult females from each Brazilian population (Rio de Janeiro, Belém and Paraná) were orally challenged with Brazilian Asian and ECSA CHIKV genotypes infected blood to determine infection rate (IR), dissemination rate (DR) and transmission rate (TR). Figure created by the author (Maria Resck) using Adobe Illustrator and Getty Images (https://www.gettyimages.pt).

Infection rate was determined by the number of females with CHIKV RNA-positive bodies from the total number that fed on the infectious blood meal. Disseminated infection rate was determined by the number of females with infected bodies that had CHIKV RNA-positive legs [42,84]. Figure 1 provides a detailed schematic overview of the experimental design.

### Statistical analyses

We analyzed the relationship between the presence or absence of CHIKV in bodies, legs, and saliva (dependent variables) of female *Ae. albopictus* and the following independent variables: CHIKV genotype (Asian and ECSA genotypes), mosquito population origin (Belém, Rio de Janeiro and Paraná) and days post-infection (dpi, 3, 5 and 13). Exploratory analyses were performed by constructing contingency tables, figures, using chi-square tests and univariate logistic models to analyze the overall relationship between each dependent variable and each of the independent variables. Likelihood-ratio Chi-square tests were used to test the significance of the sequential inclusion of the independent variables in binomial GLMs. We modeled this relationship using separate binomial generalized linear models: one focused on the bodies, one focused on the legs, and one focused on the saliva. We tested two-way interactions between genotype x population origin, genotype x days post-infection, and population origin x days post infections in all models, but the inclusion of the interactions did not incur in statistical significance. Models were evaluated via Akaike Information Criterion (AIC) to compare models with and without interactions. AIC provides a measure of the relative quality of statistical models by balancing goodness of fit and model complexity. Likelihood-ratio Chi-square tests were used to test the significance of the sequential inclusion of the independent variables in binomial GLMs. We also analyzed the relationship between viral titers of bodies, legs, and saliva and the main effects using a Gaussian Linear Model. All analyses were done using R [94] and RStudio [95].

## Results

### Chikungunya virus infection by *Ae. albopictus* population origin, genotype and days post-infection

A total of 305 and 340 *Ae. albopictus* were tested following ingestion of ECSA and Asian genotypes of chikungunya virus, respectively, across the geographic populations and three periods during the incubation.

To investigate the dynamics of CHIKV infection in *Ae. albopictus*, we measured body positivity rates, defined as the proportion of mosquitoes with detectable viral infection at 3-, 5-, and 13-days post-infection (DPI) for both Asian and ECSA genotypes.

**Fig 2:**
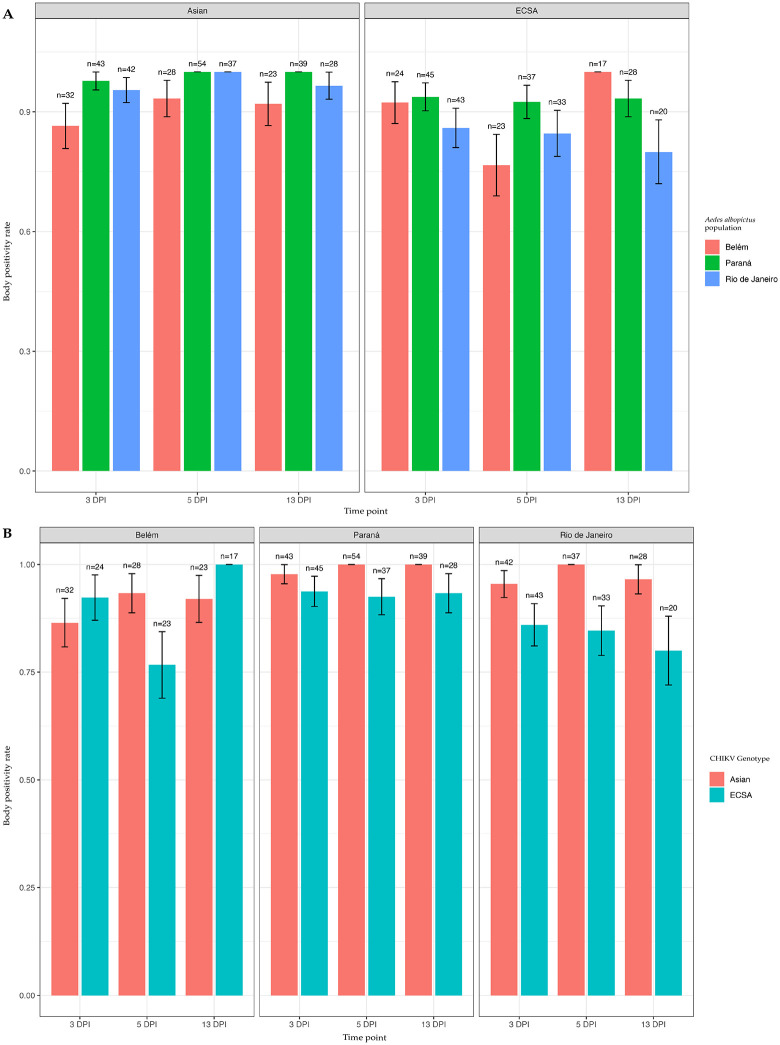
CHIKV infection rate in *Ae. albopictus* bodies across three time points (3-, 5-, and 13-days post-infection). **(A)** Infection rates separated by virus genotype (Asian and ECSA), comparing the three mosquito populations (Belém, Paraná, and Rio de Janeiro). **(B)** Infection rates separated by mosquito population (Belém, Paraná, and Rio de Janeiro), comparing the two virus genotypes (Asian and ECSA). Error bars in both panels represent standard error of the mean.

Figure 2A illustrates heterogeneity in positivity curves among Brazilian mosquito populations. For the Asian CHIKV genotype, the *Ae. albopictus* Belém population (red line) shows a relatively low positivity rate at 3 DPI, a substantial increase at 5 DPI, and a slight decrease at 13 DPI (positivity rate and lower and upper 95% Confidence Intervals; respectively 0.865 [0.809, 0.921]; 0.933 [0.888, 0.979]; and 0.92 [0.866, 0.974]). This suggests a slower initial infection establishment in this population, with peak viremia around 5 DPI. In contrast, the Paraná population (green line) exhibits high positivity rates as early as 3 DPI (0.92 [0.866, 0.974]; 1 for 5 and 7 DPI), remaining relatively stable across time points, indicating greater susceptibility or efficient early viral replication. The Rio de Janeiro population (blue line) displays an intermediate pattern, with a moderate initial positivity rate at 3 DPI (0.955 [0.923, 0.986]), an increase at 5 DPI (similar to Belém; Fig 2A), and a subsequent decrease at 13 DPI (0.966 [0.932, 0.999]), approaching initial levels. This late decrease may reflect viral clearance or lower persistence of detectable infection. Error bars indicate greater variability in the Belém and Rio de Janeiro populations, particularly at extreme time points, suggesting a more homogeneous response in the Paraná population.

Figure 2B presents CHIKV body positivity rates by mosquito population. Specifically, in the Belém population, the ECSA genotype shows heterogeneous positivity rates when compared to the Asian genotype, suggesting a varying susceptibility and replication (0.923 [0.871, 0.975]; 0.767 [0.689, 0.844]; respectively for 3, 5 and 7 DPI). The Paraná population has lower positivity rates for ECSA when compared to Asian genotype at all time points (0.938 [0.903, 0.972]; 0.925 [0.883, 0.967]; 0.933 [0.888, 0.979]). In the Rio de Janeiro population, the ECSA genotype show decreasing positivity from 3 to 5 DPI (respectively 0.86 [0.811, 0.909] and 0.846 [0.788, 0.904]) and a larger decrease at 13 DPI (0.8 [0.72, 0.88]), with the ECSA genotype showing consistently lower positivity rates when compared to the Asian genotype, especially at 3 and 5 DPI. These observations underscore the complex interaction between virus genotype and mosquito population in determining CHIKV infection dynamics.

Despite the observed variability in descriptive patterns (Fig 2A and 2B), the generalized linear model (GLM) analysis revealed no significant effect of time (days post-infection), population, or virus genotype on CHIKV positivity rates (Table 1; ANOVA results: *p* > 0.05 for all factors). This indicates that, on average, bodies of female *Ae. albopictus* populations from the tested populations tends to have a high infection rate despite origin, with most of the groups having more than 80% of infected individuals as early as 3 DPI, showing a rapid viral replication in the midgut.

**Table 1:** ANOVA results for the effect of Time Post-Infection, population, and virus genotype on CHIKV infection (body) and dissemination (legs) in *Ae. albopictus.*

Effects	LR Chisq	Df	*P* - value	Significance
**Bodies**				
Time point	0.7058	2	0.70265	
Population	1.8719	2	0.39222	
Virus genotype	3.4475	1	0.06335	
**Legs**				
Time point	33.424	2	5.521e-08	***
Population	8.528	2	0.01406	*
Virus genotype	0.103	1	0.74773	
**Saliva**				
Time point	0.93591	2	0.6263	
Population	1.34636	2	0.5101	
Virus genotype	0.00768	1	0.9302	

The viral titers in the analyzed bodies significantly associated with time post-infection (*p* = 0.003) and viral genotype (*p* = 0.0003). A significant three-way interaction (time_point × population × virus_genotype) was observed (*p* = 0.027), indicating that the effect of the viral genotype on the titer is not constant and depends on the specific combination of time and population (Table 2).

**Table 2:** ANOVA results for the effect of time post-infection, population, and virus genotype on CHIKV body viral titers in *Ae. albopictus.*

Effects	LR Chisq	Df	*P* - value	Significance
**Body viral titer (log10)**				
Time point	11.5657	2	0.0030799	**
Population	0.8406	2	0.6568430	
Virus genotype	12.6641	1	0.0003727	***
**Interactions**				
Time point X population	5.0888	4	0.2783080	
Time point X virus genotype	1.8128	2	0.4039755	
Population X virus genotype	7.3164	2	0.0257794	*
Time point X virus genotype X population	10.8909	4	0.0278179	*

Significance Codes: 0 '***' 0.001 '**' 0.01 '*' 0.05 '.' 0.1 ' ' 1

The Estimated Marginal Means (EMMs) decomposition showed that the Asian – ECSA contrast was positive in most time × population combinations; that is, the Asian genotype exhibited higher viral titers than ECSA in the majority of the evaluated treatments. In Belém, Asian titers were higher than ECSA at 3 DPI (*p* = 0.0004) and 5 DPI (*p* = 0.0045), but not at 13 DPI (*p* = 0.221). In Paraná, there was no detectable difference at 3 DPI (*p* = 0.121); however, Asian was substantially higher than ECSA at 5 DPI (*p* < 0.0001) and 13 DPI (*p* < 0.0001), consistent with the evidence of a time × genotype interaction in this population. In Rio de Janeiro, Asian was higher than ECSA at 3, 5, and 13 DPI (*p* < 0.0001 for all comparisons), suggesting a strong and persistent genotype effect in this population (S1 Fig).

### Chikungunya virus dissemination by *Ae. albopictus* population origin, genotype and days post-infection

**Fig 3:**
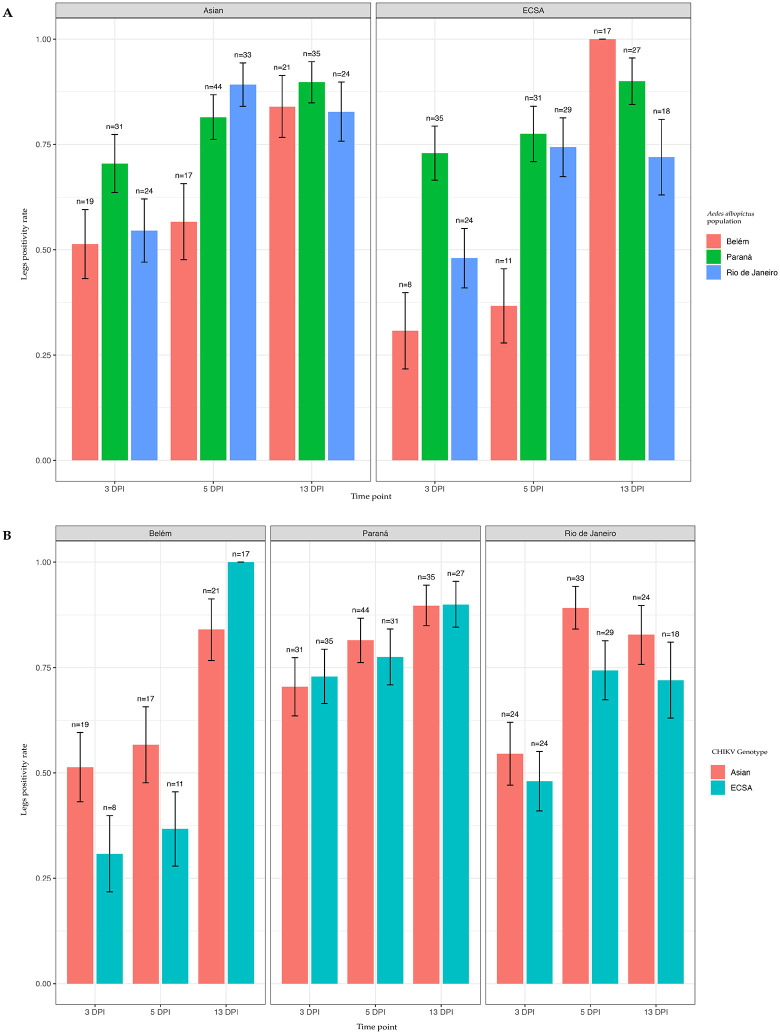
CHIKV dissemination in *Ae. albopictus* legs across three time points (3-, 5-, and 13-days post-infection). **(A)** Dissemination rates separated by virus genotype (Asian and ECSA), comparing the three mosquito populations (Belém, Paraná, and Rio de Janeiro). **(B)** Dissemination rates separated by mosquito population (Belém, Paraná, and Rio de Janeiro), comparing the two virus genotypes (Asian and ECSA). Error bars in both panels represent standard error of the mean.

Analysis of CHIKV positivity in *Ae. albopictus* mosquito legs revealed a distinct pattern compared to that observed in the mosquito bodies (Fig 3A and 3B). Generalized linear modeling demonstrated that time post-infection and mosquito population were significant factors affecting positivity rates in the legs (time point: *p* < 0.001; population: *p* = 0.014) (Table 1). There was a significant increase in positivity from 3 DPI to 5 DPI *(p* = 0.00116) and a further significant increase from 5 DPI to 13 DPI (*p* < 0.001), indicating a progressive dissemination of the virus in the legs over time, a crucial step for potential transmission. Mosquito population also played a role, with the Paraná population showing a significantly higher positivity rate than the Belém population (*p* = 0.00342), suggesting potential differences in susceptibility or dissemination efficiency between these mosquito populations. In contrast, the virus genotype (Asian or ECSA) did not significantly influence the overall positivity in the legs (*p* = 0.74791), suggesting a similar capacity for both genotypes to disseminate to this tissue among different geographic populations of *Ae. albopictus*.

The viral titers in the analyzed legs significantly correlated with both time point and viral genotype. Notably, the three-way interaction was significant (*p* = 0.048), demonstrating a complex dynamic where each population and genotype varies significantly over time (Table 3). According to the Estimated Marginal Means (EMMs) analysis, the Asian–ECSA contrast proved to be strongly context-dependent. In Belém, the Asian genotype exhibited higher titers than ECSA at 5 DPI, though no detectable difference was found by 13 DPI. In Paraná, Asian titers were significantly higher at 3 and 5 DPI but not at 13 DPI, notably without robust evidence of a time x genotype interaction within this specific population. Conversely, in Rio de Janeiro, the time x genotype interaction was significant; the Asian–ECSA contrast was most pronounced at 5 DPI and remained significant at 13 DPI, after showing no significant difference at 3 DPI (S2 Fig). These log_10_ differences reflect a significant biological fold-change, equivalent to a ten- to one-hundred-fold increase in viral titers.

**Table 3:** ANOVA results for the effect of time post-infection, population, and virus genotype on CHIKV legs viral titers in *Ae. albopictus.*

Effects	LR Chisq	Df	*P* - value	Significance
**Leg viral titer (log10)**				
Time point	9.6809	2	0.007904	**
Population	10.4529	2	0.005372	**
Virus genotype	3.3926	1	0.065488	
**Interactions**				
Time point X population	6.3591	4	0.173884	
Time point X virus genotype	5.1372	2	0.076644	
Population X virus genotype	0.9749	2	0.614204	
Time point X virus genotype X population	9.5708	4	0.048312	*

Significance Codes: 0 ‘***’ 0.001 ‘**’ 0.01 ‘*’ 0.05 ‘.’ 0.1 ' ' 1

### Chikungunya virus saliva infection by population origin, genotypes and days post-infection

**Fig 4:**
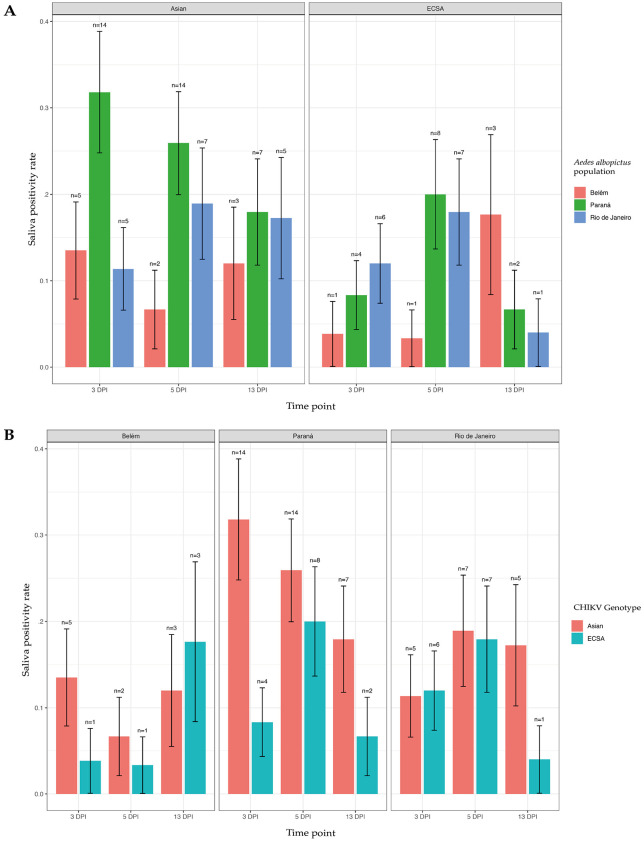
CHIKV infection rate in *Ae. albopictus* saliva across three time points (3-, 5-, and 13-days post-infection). **(A)** Infection rates separated by virus genotype (Asian and ECSA), comparing the three mosquito populations (Belém, Paraná, and Rio de Janeiro). **(B)** Infection rates separated by mosquito population (Belém, Paraná, and Rio de Janeiro), comparing the two virus genotypes (Asian and ECSA). Error bars in both panels represent standard error of the mean.

Figure 4 shows positivity rates in saliva collected from the studied Brazilian *Ae. albopictus* populations. Overall, positivity rates in saliva were much lower than those observed in bodies and legs (Figs 2–4). For both CHIKV genotypes, the Belém population showed a low positivity rate at all tested DPIs, with mean values never surpassing a rate of 0.2 (Asian: 0.135 [0.0789, 0.191]; 0.0667 [0.0211, 0.112]; and 0.12 [0.0550, 0.185]; ECSA: 0.0385 [0.000747, 0.0762]; 0.0333 [0.000560, 0.0661]; and 0.176 [0.0840, 0.269]). The Paraná population showed higher positivity rates for the Asian genotype (0.318 [0.248, 0.388]; 0.259 [0.200, 0.319]; and 0.179 [0.118, 0.241]) and a mixed profile for the ECSA genotype when comparing the DPIs (0.0833 [0.0434, 0.123]; 0.2 [0.137, 0.263]; and 0.0667 [0.0211, 0.112]). Finally, the Rio de Janeiro population showed a heterogeneous infection pattern for both Asian (0.114 [0.0658, 0.161]; 0.189 [0.125, 0.254]; and 0.172 [0.102, 0.243]) and ECSA (0.12 [0.0740, 0.166]; 0.179 [0.118, 0.241]; and 0.04 [0.000808, 0.0792]) genotypes for DPIs. Model results showed no significant effect of time (days post-infection), population origin and virus genotype, which shows that, in general, all studied populations tended to show a similar saliva infection rate despite origin, virus genotype and how many days elapsed since ingestion of CHIKV infected blood.

For the saliva viral titers, no significant effects were detected for time (*p* = 0.725) or population (*p* = 0.183). However, a robust genotype effect was observed (*p* < 0.0001): the ECSA genotype presented saliva titers approximately 1.27 log_10_ units lower than the Asian genotype. This corresponds to a biological difference of approximately 19-fold (fold-change of 10^1.273^) in viral titer magnitude, on average, while adjusting for time and population (S3 Fig). These results should be interpreted with caution, as the high rate of missing observations for saliva reduced the statistical power to detect finer variations between treatments.

Taken together, these findings support the hypothesis that genotypic differences are detectable across multiple tissues. However, spatiotemporal heterogeneity (population and time) is more pronounced during the infection and dissemination phases (body and legs). In contrast, at the final stage closest to potential transmission (saliva), the statistical inference is dominated by a CHIKV genotype effect and remains heavily constrained by the limitations of the available sampling design.

## Discussion

### Infection

*Aedes albopictus*, introduced in Brazil in the 1980s, is now well-established and widespread across the country. In this study we compared the vector competence of Brazilian populations of *Ae. albopictus* for two strains of chikungunya virus (CHIKV), Asian and ECSA, responsible for outbreaks in 2014 in Oiapoque, Amapá and Feira de Santana, Bahia regions, respectively [51,52]. Our investigation revealed distinct patterns across infection, dissemination, and transmission stages among the three Brazilian *Ae. albopictus* populations but showed similar vector competence for both Brazilian viral strains. Despite observed descriptive heterogeneity in body positivity rates among the three Brazilian *Ae. albopictus* populations (Belém, Paraná, and Rio de Janeiro) and between Asian and ECSA CHIKV genotypes (Fig 2A and 2B), generalized linear model (GLM) analysis indicated no statistically significant effect of time post-infection, mosquito population, or virus genotype on overall CHIKV infection rates (*p* > 0.05 for all factors; Table 1).

Our results also align with previous reports in the literature, which have demonstrated a lack of significant genetic and geographic variations in *Ae. albopictus* susceptibility to infection for CHIKV. Severini et. al. (2018) [79] for instance, demonstrated that all eight Italian populations of *Ae. albopictus* tested exhibited comparable abilities to be infected by the mutated ECSA CHIKV strain (E1-226V), with no statistically significant differences observed in mean viral titers within the bodies (Kruskal Wallis test, *p* = 0.825). Fortuna et al (2018) [88] supported that same information with one population of Italian *Aedes albopictus* for two strains of chikungunya virus (CHIKV), with and without E1:A226V mutation. More broadly, these results underscore the established role of *Ae. albopictus* as a highly competent vector for CHIKV across diverse genotypes and geographical origins. Several studies, consistently report its efficient infection capabilities, suggesting similar and high permeability of the midgut infection barrier [28,42,49,79,80,85–88,90,91].

The lack of a statistically significant difference suggests that, once exposed to an infectious blood meal, the midgut barrier in these Brazilian *Ae. albopictus* populations is generally highly permissive to both Asian and ECSA CHIKV genotypes. This rapid and high initial infection establishment, with over 80% of individuals infected as early as 3 days post-infection (DPI) regardless of origin, implies a highly efficient process of viral entry and replication within the midgut cells. It is possible that the descriptive variations observed (e.g., Belém’s slower initial rise for Asian genotype) represent subtle kinetic differences that do not ultimately translate into significant overall differences in the proportion of infected mosquitoes over the 13-day period. The unique kinetic pattern observed in Belém — characterized by a delayed infection peak at 5 — could reflect slower viral replication kinetics within the midgut. This potential ‘saturation effect’ by 3–5 DPI may mask early variations that could be more apparent at lower viral doses or earlier time points. Given the widespread distribution of *Ae. albopictus* across Brazil, this uniform susceptibility to high-dose infection underscores the species’ significant potential to facilitate the local spread of CHIKV outbreaks, regardless of the circulating genotype.

### Dissemination

In contrast to infection, CHIKV dissemination to the legs was significantly influenced by both time post-infection (*p* < 0.001) and mosquito population (*p* = 0.014; Table 1). We observed a strong and progressive increase in leg positivity over time, with significant increases for each subsequent period measured following ingestion of CHIKV infected blood. This highlights the time-dependent nature of viral spread within the mosquito. This progressive increase in disseminated infection is consistent with the general understanding of arbovirus replication kinetics within the mosquito vector, where the virus must overcome the midgut barrier, replicate, and then spread systemically to reach secondary tissues like the legs before ultimately infecting the salivary glands. Furthermore, the Paraná *Ae. albopictus* population exhibited a significantly higher dissemination rate than the Belém population (*p* = 0.00342), suggesting a potentially greater intrinsic susceptibility or more efficient viral spread within the Paraná mosquitoes. Notably, neither Asian nor ECSA CHIKV genotypes significantly impacted overall dissemination rates (*p* = 0.748), implying similar capacities for both genotypes to disseminate to peripheral tissues. These observations suggest that populations of *Ae. albopictus* from Paraná may exhibit a less effective midgut escape barrier or a greater magnitude of viral replication within the midgut — or a combination of both — compared to other Brazilian populations tested, and future studies should investigate the relative contribution of each mechanism.

These observed patterns of dissemination are supported by and expand upon findings from other comparative studies on *Aedes* vector competence for CHIKV. For instance, Honório et al 2018 [42] conducted studies using the Asian genotype of CHIKV across Brazilian and Florida *Ae. aegypti* and *Ae.* *albopictus* populations, and their results also demonstrated a significant increase in disseminated infection over time, consistent with our findings on the time-dependent nature of viral spread. Honório et al. 2018 [42] primarily focused on the Asian CHIKV genotype, however, in contrast to our observation of a significant population effect on dissemination (as observed with the Paraná vs. Belém populations), these authors also found no significant effect of mosquito population origin on viral dissemination rates in their study. Similarly, Severini et al 2018 [79] found that Italian *Ae. albopictus* populations showed no statistically significant differences in dissemination rates or mean viral titers in legs and wings (Kruskal Wallis test, *p* = 0.609), despite high overall dissemination. This discrepancy highlights potential differences in the specific populations tested, experimental conditions, or the statistical power to detect such effects across studies.

Vega-Rúa et al. (2020) [96] further elucidated this complexity, demonstrating that the vector competence of *Ae. albopictus* populations for CHIKV, including both dissemination and transmission efficiencies, is significantly influenced by their demographic history and genetic ancestry. Their work, which investigated different CHIKV strains (Asian, ECSA, ECSA E1-226V), revealed complex genotype-by-genotype interactions where mosquito genetic lineages and specific viral strains influenced competence, leading to varying dissemination and transmission efficiencies across different *Ae. albopictus* populations.

This population-specific difference in dissemination, in contrast to the uniform rates of initial infection, points to a potential bottleneck or barrier to viral spread beyond the midgut that varies among mosquito populations. Notably, neither the Asian nor ECSA genotypes significantly impacted overall dissemination rates, a finding that aligns with observations across ten countries in the Americas [28]. This suggests that while these genotypes may exhibit subtle differences in their initial midgut replication kinetics, their ability to overcome systemic barriers and reach peripheral tissues is comparable. Our results further emphasize that once a midgut infection is established, Brazilian *Ae. albopictus* is highly and equally permissive to the systemic spread of both circulating strains. This time-dependent progression of viral dissemination is a crucial biological determinant of the extrinsic incubation period (EIP), directly impacting the duration of a mosquito’s infectiousness and its overall capacity for onward transmission in the field.

### Transmission

Our finding regarding transmission was the consistently low positivity rates in saliva across all *Ae. albopictus* populations and CHIKV genotypes, which were substantially lower than those observed in bodies and legs (Fig 4). This result highlights a significant barrier in the transmission process occurring at or before the infection of the salivary glands and subsequent release of infectious virus into the saliva. Despite visual variations in the descriptive patterns, a generalized linear model revealed no significant effect of days post-infection, mosquito population, or virus genotype on saliva positivity rates. This indicates that, on average, the probability of infectious virus being present in the saliva, and thus capable of transmission, remains consistently low regardless of these factors.

The mean saliva viral titers observed in positive mosquitoes provide additional context for interpreting transmission potential. For the Asian genotype, mean saliva titers ranged from 2.53 to 4.06 log₁₀ PFUe/mL across populations and time points. Notably, the published minimum infectious dose for CHIKV in *Ae. albopictus* has been estimated at approximately 3.9 log₁₀ pfu/mL [97], suggesting that only a subset of saliva-positive mosquitoes — those at the higher end of this range — may have shed virus at levels sufficient for effective transmission. In contrast, mean saliva titers for the ECSA genotype were consistently lower across all populations and time points (1.51 to 3.72 log₁₀ PFUe/mL), falling below this threshold in most conditions, further supporting the limited transmission potential of this genotype even among saliva-positive individuals.

We observed a trend for a reduction in the proportion of mosquitoes with a saliva infection at later time points in the Belém and Paraná mosquitoes infected with the Asian genotype of CHIKV. These results are consistent with observations that saliva infection declined with the length of infection in *Ae. albopictus* from Okeechobee, Florida and Rio de Janeiro, Brazil infected with an Asian genotype of CHIKV [42]. Impaired transmission efficiency among older mosquitoes is likely attributable to virus modulation of infection as observed in other mosquitoes and arboviruses [98–100]

The progression from midgut infection to salivary gland infection involves at least two well-characterized barriers: the midgut escape barrier (MEB), which limits systemic dissemination, and the salivary gland infection barrier (SGIB), which limits viral entry and replication within salivary gland tissue. Our data suggest that the MEB is relatively permissive in all three Brazilian *Ae. albopictus* populations, given the high and rapid body infection rates (>80% at 3 DPI) and progressive dissemination to the legs over time. However, the substantially lower saliva positivity rates — despite high dissemination — point to the SGIB as the predominant bottleneck limiting transmission. The lower saliva titers observed for the ECSA genotype compared to Asian, even among saliva-positive individuals, may further reflect genotype-specific differences in the ability to replicate within or escape from salivary gland cells, rather than simply differences in systemic viral load. The Belém population showed very low transmission potential, never surpassing a mean 0.2 positivity rate for either genotype across all time points. This might suggest an even more pronounced SGIB or a less conducive environment for viral replication within the salivary glands, although the overall lack of statistical significance for population effect on saliva positivity suggests a generalized challenge for transmission across all tested *Ae. albopictus* groups. Regardless of similar transmission rates observed among populations, greater absolute numbers of disseminated infections (i.e., from the total number of mosquitoes ingesting infected blood) exhibited by the Paraná population of *Ae. albopictus* is predicted to yield a higher risk of saliva infection relative to other populations of *Ae. albopictus* from Brazil*.*

This overall low and non-variant transmission efficiency, despite high infection and significant dissemination in some populations, contrasts sharply with the known role of *Ae. albopictus* as an important CHIKV vector in other geographical regions. For instance, Vega-Rúa et al. (2014) [28] reported transmission efficiencies reaching up to 96.7% in American populations, and Honório et al. (2018) [42] found rates as high as 82% in Brazilian *Ae. albopictus* populations (e.g., from Manguinhos, Rio de Janeiro). Furthermore, Richards et al. (2010) [80] observed the highest infection, dissemination, and transmission rates in Florida *Ae. albopictus* compared to *Ae. aegypti* and *Culex pipiens quinquefasciatus*, concluding that, *Ae. albopictus* is a competent vector of CHIKV, though they also noted a bottleneck where not all mosquitoes with disseminated infections could transmit the virus in saliva. However, the low numbers of *Ae. albopictus* females successfully reaching the final stage of the experiment can be considered an important limitation of our study. This reduced sample size in the saliva group may have limited our ability to resolve finer statistical interactions that were more apparent in the body and leg tissues.

The observed epidemiological trends of chikungunya in Brazil, marked by significant case numbers and fatalities from 2023 to 2025, underscore the critical role of vector dynamics in disease transmission. Our findings, revealing higher heterogeneity in body positivity rates among *Ae. albopictus* populations from Northern, Southeastern, and Southern Brazil exposed to locally circulating Asian and ECSA CHIKV genotypes, raise pertinent questions regarding the intrinsic competence of these specific mosquito populations under varying conditions. Factors such as diverse environmental settings or serial viral passages could influence these rates, suggesting a complex interplay between the vector, the virus, and local ecological factors. A deeper understanding of this variability is crucial for refining predictive models and developing targeted public health interventions, especially given *Ae. albopictus*’s potential as a “bridge vector” at the urban-forest interface, which could facilitate future arbovirus spill-over events and outbreaks.

While this study provides important insights into CHIKV vector competence, some considerations should be noted. The use of a relatively high viral titer and a standardized artificial feeding system, although necessary for experimental control, may not fully reflect natural infection conditions. In addition, the limited number of mosquitoes reaching the saliva analysis stage may have reduced statistical power. Despite this, the study design enabled robust comparisons across populations and genotypes.

## Supporting information

S1 Fig Mean body CHIKV viral titer (log_10_) in *Ae. albopictus* across three time points (3-, 5-, and 13-days post-infection) separated by virus genotype (Asian and ECSA), comparing the three mosquito populations (Belém, Paraná, and Rio de Janeiro). (TIFF)

S2 Fig Mean legs CHIKV viral titer (log_10_) in *Ae. albopictus* across three time points (3-, 5-, and 13-days post-infection) separated by virus genotype (Asian and ECSA), comparing the three mosquito populations (Belém, Paraná, and Rio de Janeiro). (TIFF)

S3 Fig Mean saliva CHIKV viral titer (log_10_) in *Ae. albopictus* across three time points (3-, 5-, and 13-days post-infection) separated by virus genotype (Asian and ECSA), comparing the three mosquito populations (Belém, Paraná, and Rio de Janeiro). (TIFF)
