# Supplementary figures and images for: Vector competence of Aedes albopictus from Northern, Southeastern, and Southern Brazil for locally circulating East-Central-South African and Asian genotypes of Chikungunya virus
Source: PLoS Negl Trop Dis. 2026 Jul 21;20(7):e0014522. doi: 10.1371/journal.pntd.0014522 (PMC13387517; doi:10.1371/journal.pntd.0014522)

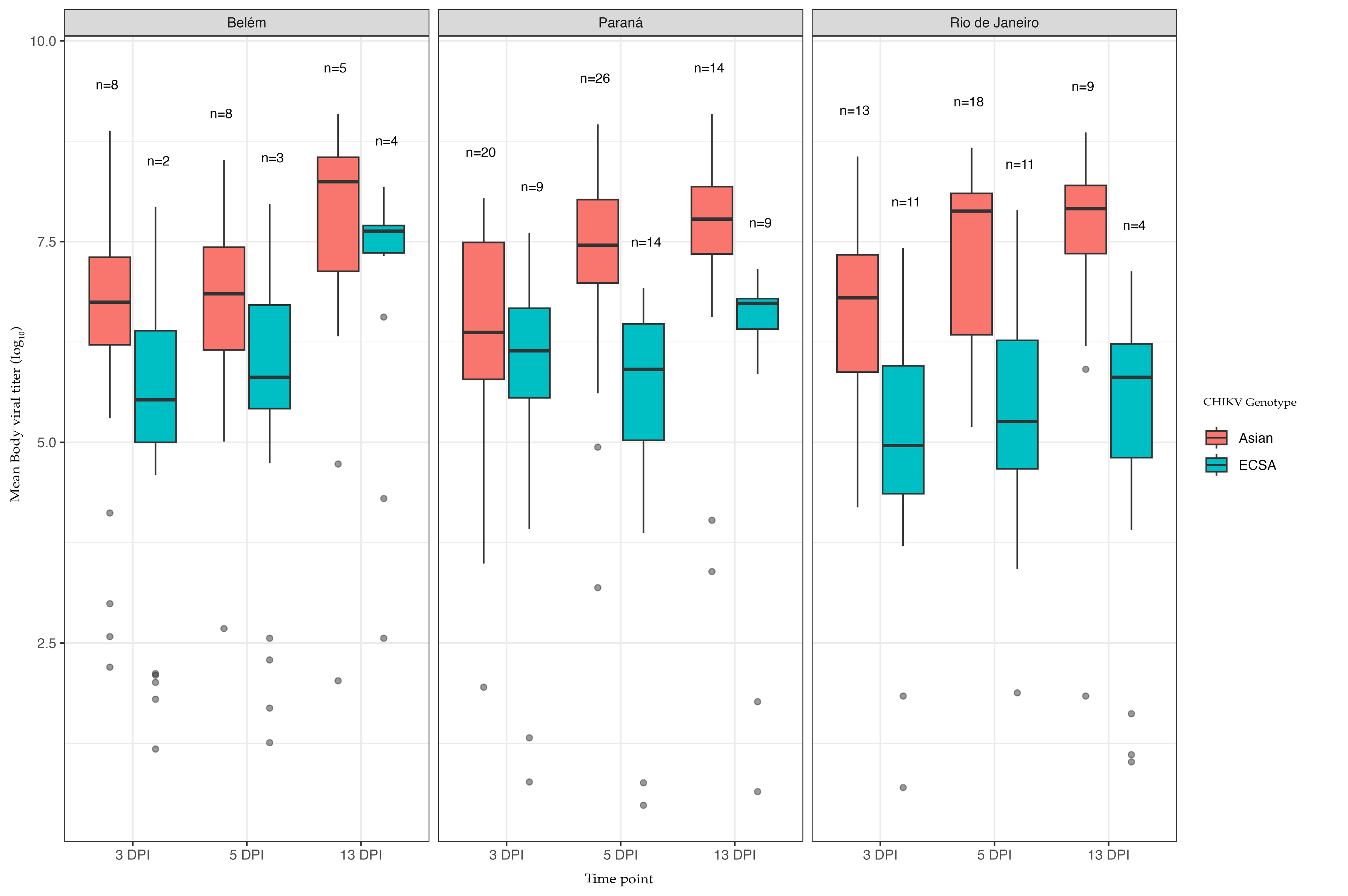

Supplement: S1 Fig — (TIFF) [file pntd.0014522.s001.tiff]

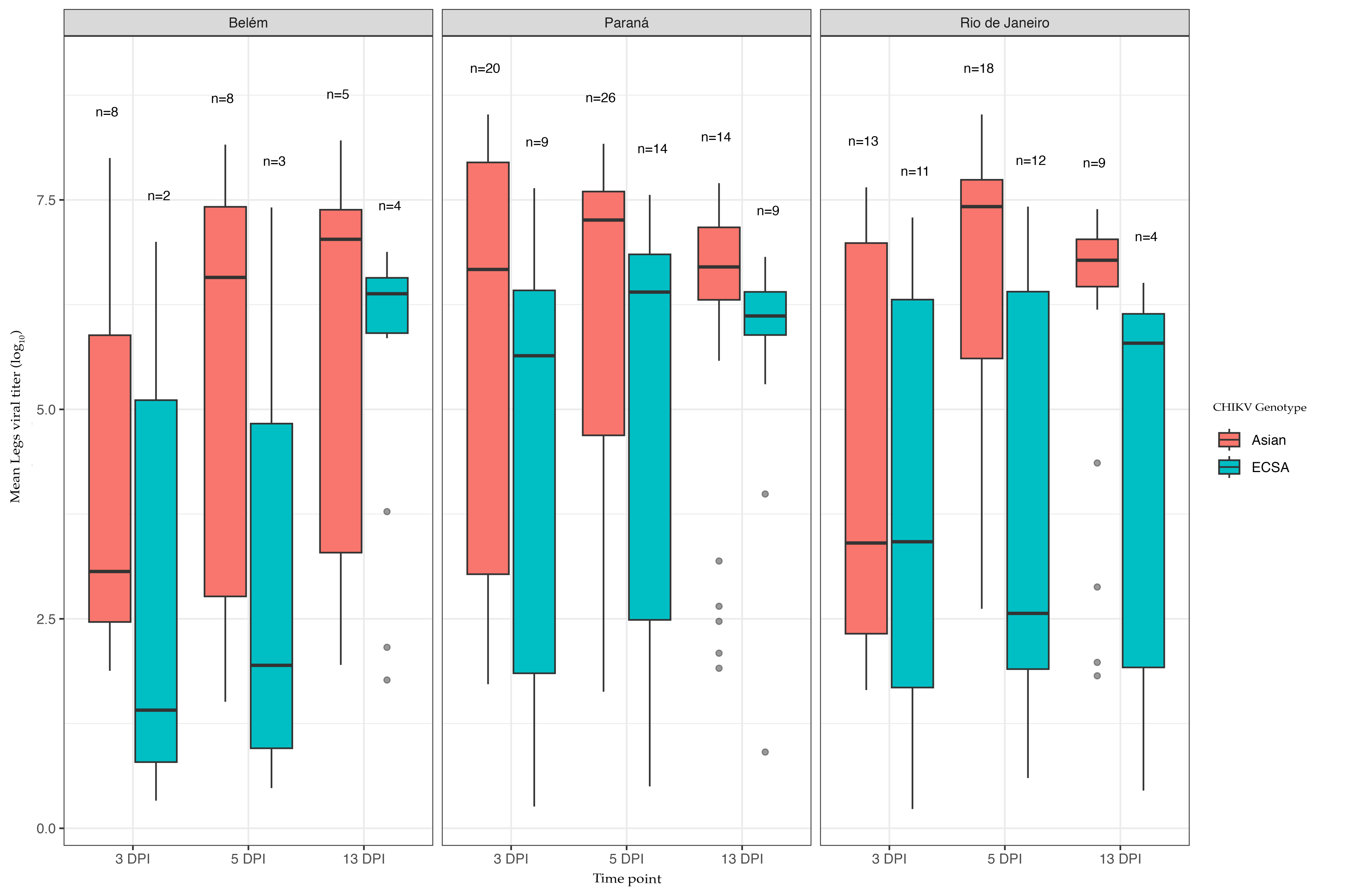

Supplement: S2 Fig — (TIFF) [file pntd.0014522.s002.tiff]

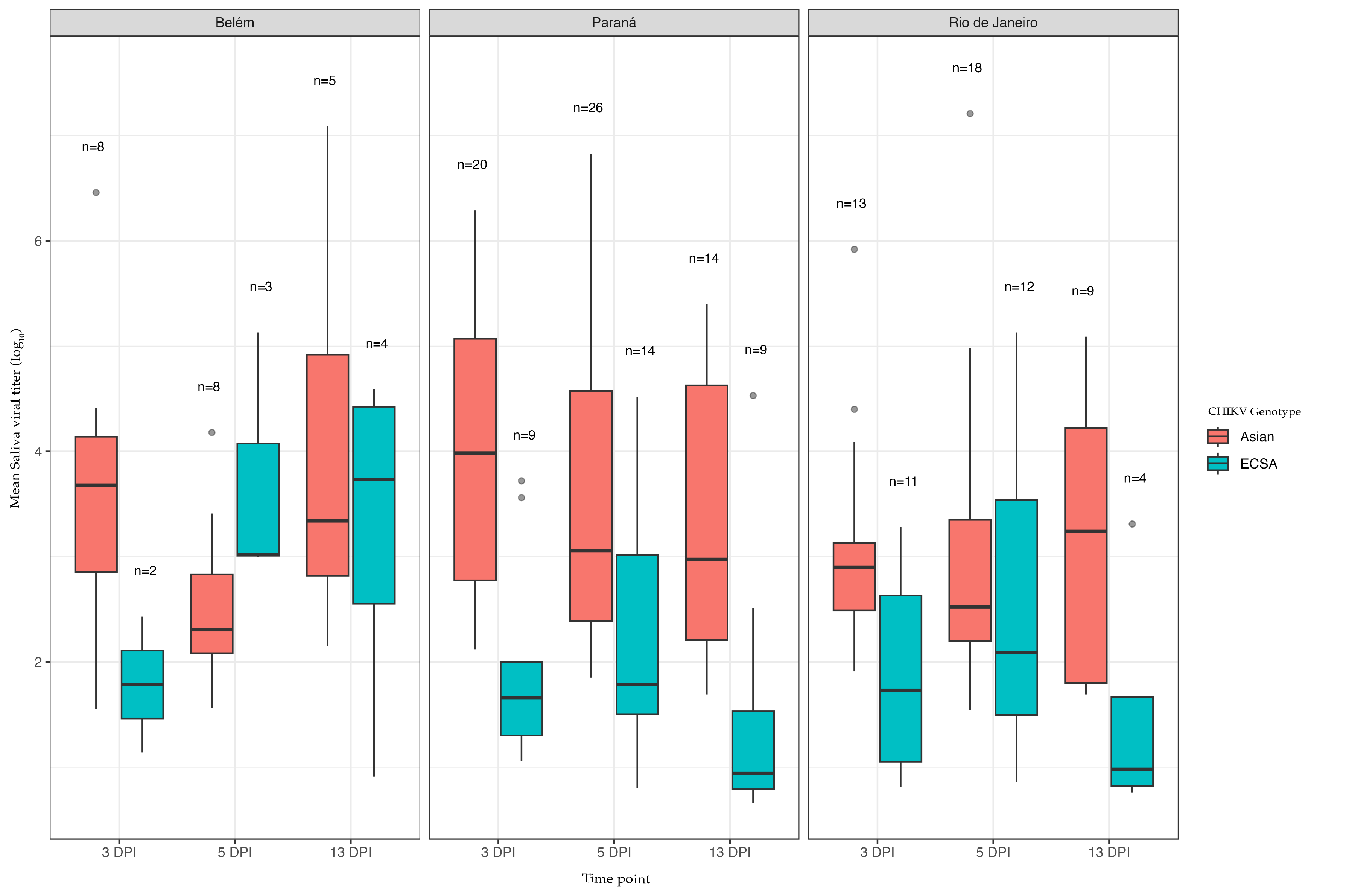

Supplement: S3 Fig — (TIFF) [file pntd.0014522.s003.tiff]
